# Beyond sarcomere genetics: proteomic insights into hypertrophic cardiomyopathy

**DOI:** 10.1007/s12551-026-01445-8

**Published:** 2026-06-06

**Authors:** Zachery R. Gregorich, Zhan Gao, Kalina J. Rossler, J. Carter Ralphe, Timothy J. Kamp, Ying Ge

**Affiliations:** 1 Department of Animal & Dairy Sciences, University of Wisconsin-Madison, Madison, WI 53706 USA; 2 Department of Cell and Regenerative Biology, University of Wisconsin-Madison, Madison, WI 53705 USA; 3 Department of Pediatrics, University of Wisconsin-Madison, Madison, WI 53705 USA; 4 Department of Medicine, University of Wisconsin-Madison, Madison, WI 53705 USA; 5 Department of Chemistry, University of Wisconsin-Madison, Madison, WI 53706 USA; 6 Human Proteomics Program, School of Medicine and Public Health, University of Wisconsin-Madison, Madison, WI 53705 USA

**Keywords:** Sarcomere genetics, Proteomic insights, Hypertrophic cardiomyopathy

## Abstract

Hypertrophic cardiomyopathy (HCM) has long been viewed as the archetypal monogenic disorder caused by pathogenic variants in the genes encoding components of the sarcomere. However, the fact that only one-third of HCM cases are genotype-positive, as well as other factors such as incomplete disease penetrance and marked phenotypic heterogeneity, challenge this reductionist view. Recent advances in mass spectrometry-based proteomics have provided new opportunities to interrogate human HCM myocardium at unprecedented depth and are reshaping our understanding of HCM pathobiology. In this mini-review, we summarize insights from both top-down and bottom-up proteomics studies showing that HCM is characterized by broad molecular remodeling across multiple cellular compartments, including the sarcomere, sarcoplasmic reticulum, cytoskeleton, mitochondria, and nucleus. Together, these studies support a model in which diverse HCM genotypes converge on shared downstream proteomic phenotypes and highlight proteomics as a powerful approach for defining disease mechanisms, modifiers, and therapeutic targets.

## Introduction

### HCM

HCM is a complex, heterogeneous disorder characterized by hypertrophy of the left ventricular free wall, and frequently the interventricular septum, that cannot be explained by abnormal loading conditions such as systemic hypertension or aortic valve stenosis (Braunwald 2025; Maron and Maron 2013). HCM is relatively common, with a prevalence of approximately 1 in 500 individuals in the general population (Maron et al. 1995), although some studies suggest the true prevalence may be as high as 1 in 200 (Semsarian et al. 2015). Patients with HCM often experience adverse outcomes, including heart failure and arrhythmias like atrial fibrillation and ventricular tachycardia. Regarding arrhythmia, HCM is a common cause of sudden cardiac death, particularly in young people (Elliott et al. 2014; Gersh et al. 2011; Maron and Maron 2016). HCM occurs across all ethnicities, sexes, and geographic regions, even though the disease is likely underrecognized in women and certain races (Shiwani et al. 2025; Wells et al. 2018).

**Fig. 1:**
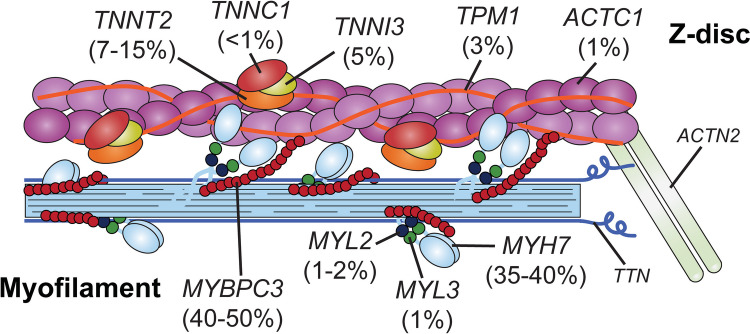
Schematic representation of the cardiac sarcomere highlighting HCM genes. Schematic shows the actin-based thin filament, consisting of cardiac actin, tropomyosin, and troponin complex (cardiac troponin I, cardiac troponin T, troponin C), and myosin-based thick filament composed of myosin heavy chain, myosin essential light chain, myosin regulatory light chain, and myosin-binding protein C. The estimated prevalence of the select HCM genes is shown in parentheses (based on information provided in reference 21). Adapted from reference 50. *TNNT2*, *TNNC1*, *TNNI3*, *TPM1*, *ACTC1*, *MYBPC3*, *MYL2*, *MYL3*, and *MYH7* encode cardiac troponin T, troponin C, cardiac troponin I, α-tropomyosin, cardiac actin, myosin-binding protein C, myosin regulatory light chain, myosin essential light chain, and myosin heavy chain, respectively. *ACTN2* and *TTN* encode α-actinin-2 and titin, respectively

Early studies of large HCM kindreds revealed an autosomal dominant pattern of inheritance (Evans 1949; Jarcho et al. 1989; Pare et al. 1961) with most causative genes encoding protein components of the sarcomere(Fig. 1). Variants in the *MYH7*gene, encoding the sarcomeric protein β-myosin heavy chain, were the first identified cause of HCM (Geisterfer-Lowrance et al. 1990). This discovery was soon followed by the identification of* TPM1 *[α-tropomyosin [(αTpm also known as Tpm1.1 (Geeves et al. 2015)] and* TNNT2 *[cardiac troponin T (cTnT)], as HCM genes, leading to the concept that HCM is a disease of the sarcomere (Thierfelder et al. 1994). This view was further reinforced by the subsequent identification of additional sarcomeric HCM genes, including *MYL3* and *MYL2*, which encode the ventricular essential (MLC-1v) and regulatory light chains (MLC-2v), respectively (Poetter et al. 1996), as well as* TNNI3 *[cardiac troponin I (cTnI)] (Kimura et al. 1997), *MYBPC3 *(myosin-binding protein C) (Niimura et al. 1998),* ACTC1 *(α-cardiac actin) (Mogensen et al. 1999), and* TNNC1 *[troponin C (TnC)] (Hoffmann et al. 2001). Together, these eight sarcomeric genes have the strongest evidence for pathogenicity and account for over 90% of genotype-positive HCM cases (Fig. 1) (Ingles et al. 2019; Lopes et al. 2024). Among them, variants in *MYBPC3* and *MYH7* are the most common (accounting for ~80% of genotype-positive cases) (Fig. 1) (Marian 2021).

### HCM—more than a disease of the sarcomere?

In recent years the long-held view of HCM as a monogenic disease caused by variants in genes encoding components of the sarcomere has been challenged (Maron et al. 2019). There are a myriad reasons for this. First, genotype-positive cases account for only one-third of reported cases (Topriceanu et al. 2024). Second, even among individuals carrying definitively pathogenic variants in sarcomeric genes, disease penetrance is frequently incomplete with phenotypic expression ranging from severe HCM to asymptomatic in so-called genotype-positive phenotype-negative individuals (Maron et al. 2011). While some of the genotype-positive phenotype-negative population may be accounted for by misclassification of benign variants as pathogenic, longitudinal analysis of HCM families suggests that many of these individuals do not manifest disease despite having the same HCM genotype as their afflicted family members (Maurizi et al. 2019). This finding reinforces the necessity for other factors (environmental, etc.) to explain disease expressivity. Third, certain structural features of the disease do not fit with what would be expected for a monogenic disease. A prime example is left ventricular hypertrophy. Despite homogeneous expression of the mutant protein, hypertrophy is asymmetric (Rowin et al. 2020). These and many other factors highlight the poor genotype–phenotype correlation in HCM and suggest that disease expressivity is likely influenced by other factors, including modifier genes outside the sarcomere (polygenicity) and regulatory mechanisms functioning upstream of genes (e.g., those regulating transcript abundance or protein activity).

### Mass spectrometry (MS)-based proteomics

MS-based proteomics holds great potential to help unravel the complex molecular mechanisms underlying HCM by enabling systematic analysis of protein expression, proteoform diversity, and post-translational modifications (PTMs) associated with disease pathogenesis. First, regarding increasing recognition of the polygenic nature of HCM (Harper et al. 2021; Tadros et al. 2021, 2025), proteomics can broadly screen protein expression to identify dysregulated pathways contributing to disease pathogenesis. On this point, proteomics offers important advantages over RNA-sequencing as the correlation between transcript and protein expression is often limited (Maier et al. 2009). Second, and perhaps more importantly, beyond simple changes in protein expression, nearly all aspects of protein biology are regulated at the level of proteoforms—a term used to describe the myriad protein species arising from genetic variants, alternative splicing, and PTMs (Aebersold et al. 2018; Smith and Kelleher 2013). PTMs are increasingly recognized for their critical roles in the development of pathological cardiac hypertrophy and heart failure (Fert-Bober et al. 2018; Lam et al. 2016; Yan et al. 2019).

**Fig. 2:**
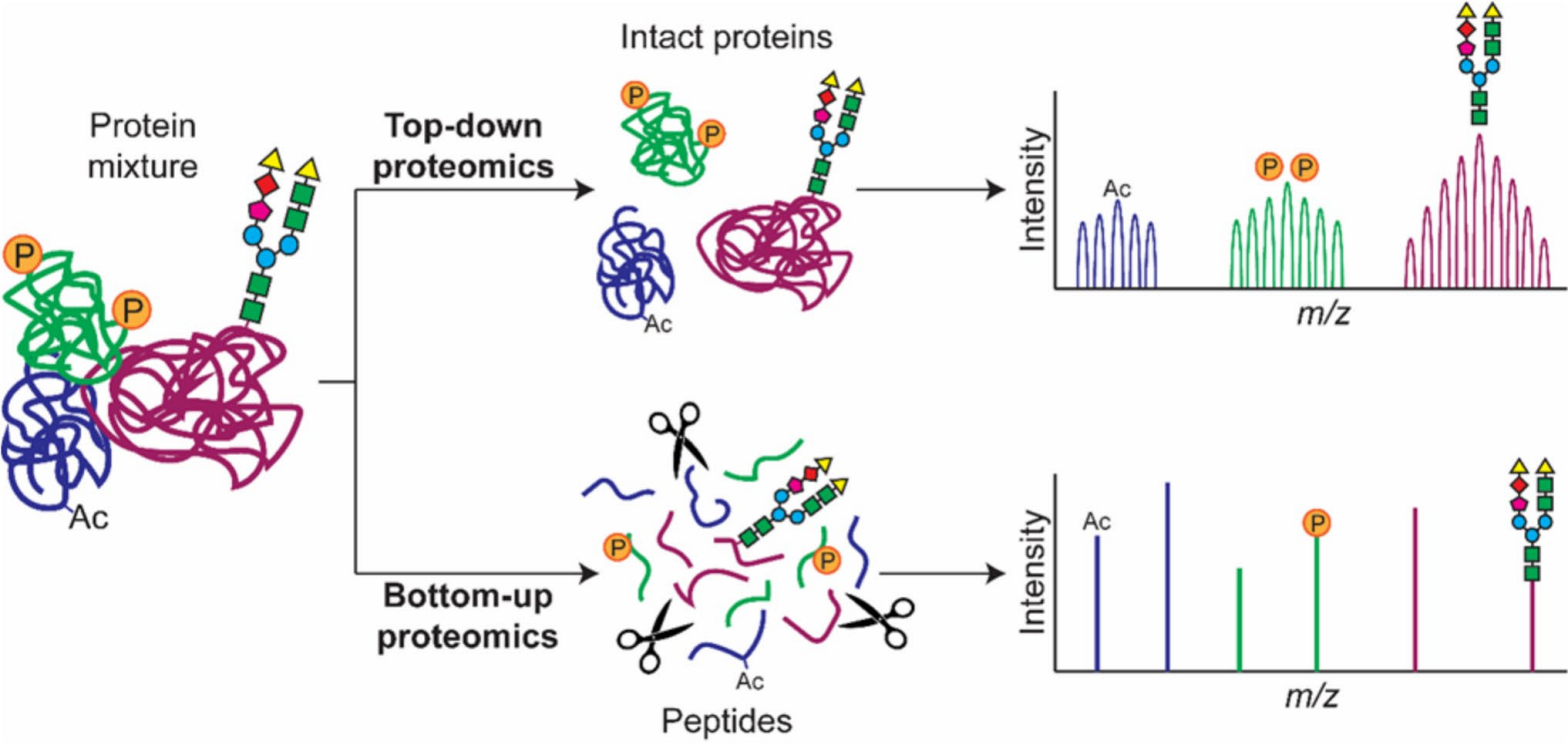
Illustration showing top-down and bottom-up proteomics workflows. In bottom-up proteomics, proteins are digested using an enzyme such as trypsin and the resulting peptides are analyzed by liquid chromatography-mass spectrometry to provide sequence information and identify post-translational modifications. Conversely, in top-down proteomics, intact proteins are analyzed providing an overview of proteoforms (term referring to the plethora of protein products produced from a single gene through genetic variants, alternative splicing, and post-translational modifications). Following intact protein analysis, proteoforms of interest can be selectively fragmented to obtain sequence information and localize sequence variants and post-translational modifications. Adapted from reference 42

Proteomics is commonly performed using two primary approaches: bottom-up proteomics (BUP) and top-down proteomics (TDP) (Fig. 2). In BUP, proteins are first digested into peptides with an enzyme like trypsin and the resulting peptides are separated, often using liquid chromatography, before MS analysis (Fig. 2) (Aebersold and Mann 2003, 2016; Guo et al. 2025; Zhang et al. 2013). Sequence information obtained from the analyzed peptides is subsequently used to identify and determine the relative abundances of the proteins present in the original sample. Conversely, in TDP, intact proteins are analyzed rather than peptides, providing a “bird’s eye” view of the proteoforms present in the sample (Fig. 2) (Roberts et al. 2024; Toby et al. 2016). Each method has advantages and disadvantages for protein analysis. While BUP boasts high-throughput and deep proteome coverage with well-developed software packages for data analysis, sequence coverage is often limited, which complicates proteoform-level analysis (Nesvizhskii and Aebersold 2005). Moreover, BUP suffers intrinsic limitations in “peptide-to-protein inference,” resulting in a loss of information and molecular connectivity when mapping sequence variations and PTMs (Plubell et al. 2022; Smith and Kelleher 2018). On the other hand, although TDP generally has lower throughput and limited proteome coverage due to challenges associated with intact protein solubility and separation, it is ideally suited for the analysis of proteoforms and new strategies have been developed to address the challenges in TDP (Roberts et al. 2024). Recent proteomics studies employing these approaches have provided important insights into the proteins and pathways outside the sarcomeres that contribute to HCM pathobiology, as highlighted in this review.

## Insights into HCM revealed by MS-based proteomics

### TDP—dysregulated proteoforms in the sarcomeres, as well as sarcoplasmic reticulum, cytoskeleton, mitochondria, and nucleus

**Fig. 3:**
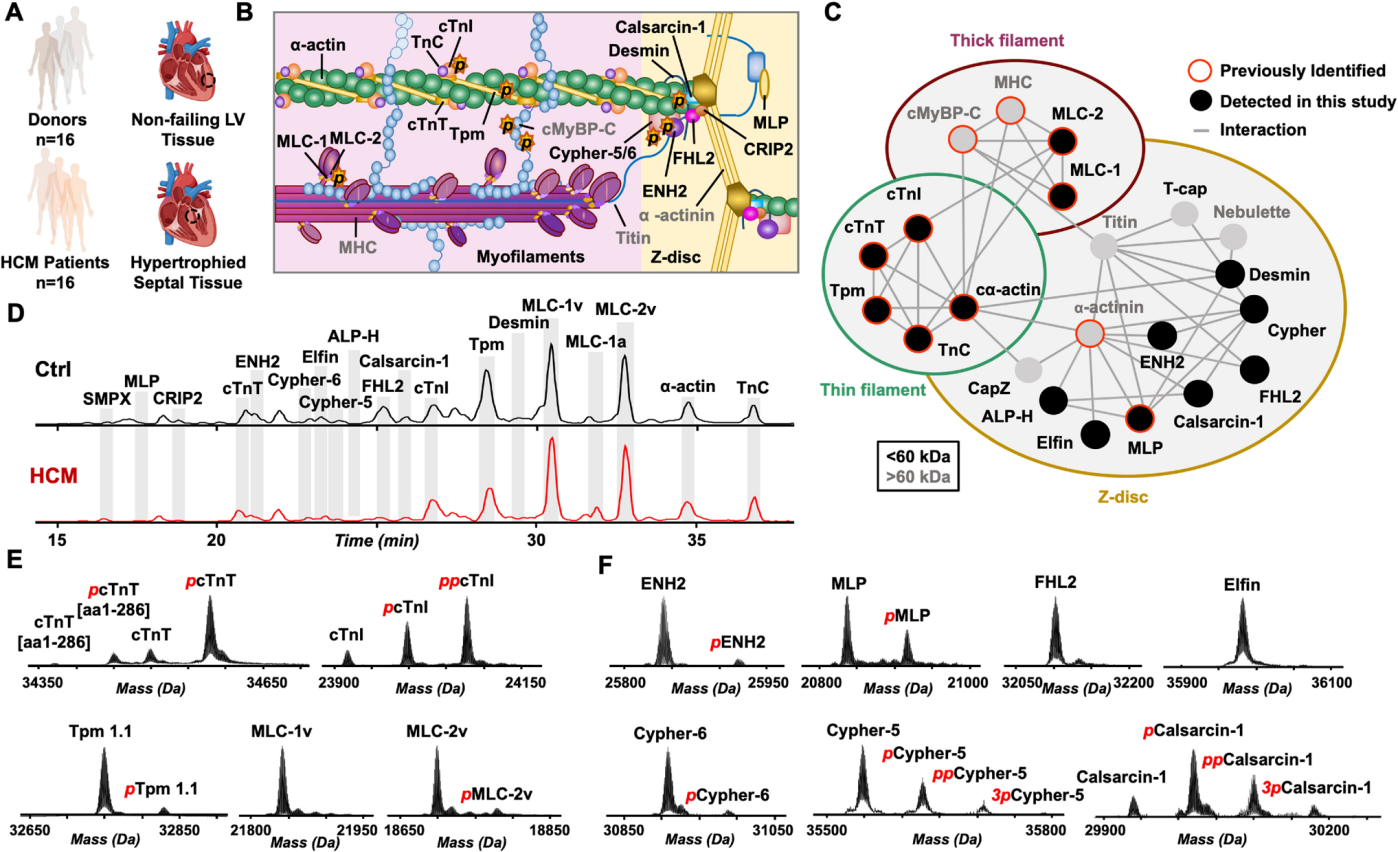
A complex sarcomeric proteoform landscape. **A** Control tissue from left ventricle (LV) of nonfailing donor hearts (Ctrl, *n* = 16) and HCM tissues procured via surgical septal myectomy procedure (HCM, *n* = 16). **B** Schematic representation of cardiac sarcomere, consisting of thin (green) and thick filaments (pink) flanked by Z-disc. **C** Sarcomeric protein interactome showing a complex network of interactions between myofilament and Z-disc proteins. **D** Representative base peak chromatograms showing separation and detection of major sarcomeric proteins by liquid chromatography-MS for Ctrl and HCM tissues. **E**, **F** Representative deconvoluted mass spectra showing the proteoforms of (**E**) myofilament and (**F**) Z-disc proteins. Figure and legend adapted from reference 47

Given the long-standing view of HCM as a disease of the sarcomere, our initial studies focused on interrogating proteoform alterations in the sarcomeres (Tucholski et al. 2020). TDP was employed to comprehensively characterize sarcomeric proteoforms in septal myectomy samples collected from patients with obstructive HCM and nonfailing controls free of cardiovascular disease (Tucholski et al. 2020). Enabled by a robust TDP workflow featuring efficient chromatography and high-resolution MS, this TDP analysis revealed a complex sarcomeric proteoform landscape arising from alternatively spliced protein isoforms and combinatorial PTMs (Fig. 3) (Tucholski et al. 2020). Among the detected proteins were major myofilament components, including cTnT, cTnI, TnC, Tpm isoforms, MLC-1 isoforms [MLC-1v and the atrial essential light chain (MLC-1a)], MLC-2v, and α-actin isoforms (Fig. 3) (Tucholski et al. 2020). Importantly, the analysis extended beyond canonical myofilament proteins to achieve proteoform-level characterization of multiple Z-disc proteins, such as FHL2, ALP-H, elfin, cypher-5, cypher-6, and calsarcin-1, revealing that proteoform complexity in HCM spans the broader sarcomeric network (Fig. 3) (Tucholski et al. 2020). Moreover, ultrahigh-resolution Fourier transform ion cyclotron resonance MS enabled direct sequence characterization of cypher-5, cypher-6, and calsarcin-1, further highlighting the strength of TDP for resolving structurally and functionally important Z-disc proteoforms in human myocardium (Tucholski et al. 2020).

**Fig. 4:**
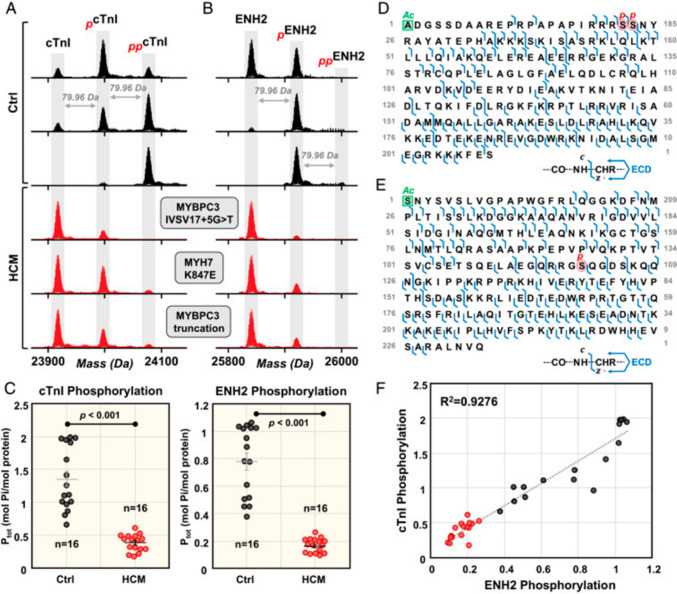
Coordinated decrease in cTnI and ENH2 phosphorylation in HCM tissues. Representative deconvoluted mass spectra for (**A**) cTnI and (**B**) ENH2 from donor hearts (black) and HCM tissues (red). Mono- and bis-phosphorylation are denoted by red p and pp, respectively. **C** Total protein phosphorylation (Ptot) calculated by mol Pi/mol protein for cTnI and ENH2 in Ctrl (*n* = 16) and HCM (*n* = 16). Horizontal bars represent the mean of the group and error bars represent SEM in gray for Ctrl and black for HCM. Groups were considered significantly different at *P* < 0.05. **D** Localization of cTnI phosphorylation to Ser22/23 and (**E**) localization of ENH2 phosphorylation to Ser118 by electron capture dissociation. **F** Linear correlation between cTnI phosphorylation and ENH2 phosphorylation (R^2^ = 0.9276). Figure and legend adapted from 47

Quantitative TDP analysis revealed alterations in the proteoforms of proteins localized to both the myofilaments and Z-discs from HCM septal myectomy tissues compared with nonfailing donor hearts (Tucholski et al. 2020). Significant differences in the relative abundances of cTnT, cTnI, αTpm, and MLC-2v proteoforms were detected in HCM hearts compared to nonfailing controls that equated to a decrease in the total phosphorylation of cTnI, αTpm, and MLC-2v, whereas cTnT phosphorylation was increased (Tucholski et al. 2020). Furthermore, we identified alterations in enigma homolog 2 (ENH2) proteoforms reflecting a decrease in the total phosphorylation of the protein (Tucholski et al. 2020). Interestingly, the observed decrease in ENH2 phosphorylation in HCM correlated well with that in cTnI phosphorylation (Fig. 4), suggesting concerted dysregulation of myofilament and Z-disc proteoforms in HCM (Tucholski et al. 2020). The identified site of phosphorylation in ENH2, Ser118, is located within a consensus PKA phosphorylation motif (RRXS/T) and in vitro phosphorylation confirmed phosphorylation of this site by PKA (Tucholski et al. 2020). Given that the sites phosphorylated in cTnI (Ser22/23) are also known substrates of PKA (Layland et al. 2005), these findings suggest dysregulation of PKA-mediated signaling in HCM. Importantly, the detected proteoform alterations were independent of patient genotype with seven patients with* MYBPC3* variants, one with an *MYH7 *variant, and eight of unknown genotype, all displaying similar proteoform alterations. This finding is significant as it suggests that the manifestation of obstructive HCM coalesces at the proteoform level despite distinct HCM genotypes (Tucholski et al. 2020).

**Fig. 5:**
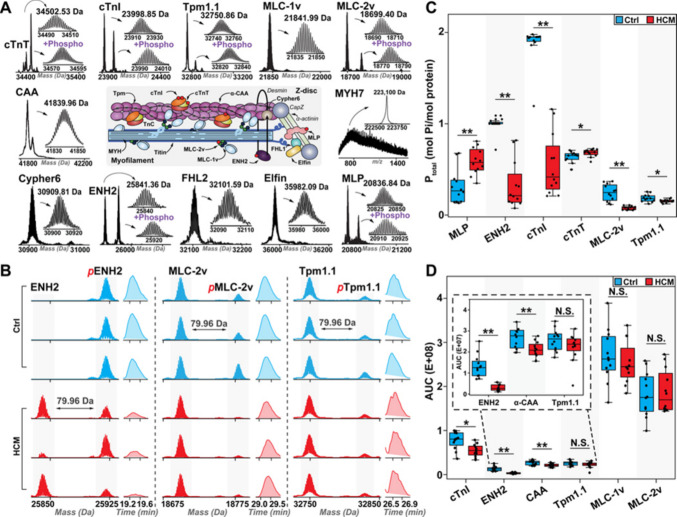
Sarcomeric proteoform alterations in hypertrophic cardiomyopathy (HCM). **A** A schematic representation of the cardiac sarcomere, consisting of the myofilament (left) and Z-disc (right), with representative deconvoluted mass spectra highlighting detected proteoforms. **B** Representative deconvoluted mass spectra (left, y axis is normalized to the peak with the highest signal intensity in each mass spectrum) showing examples of proteoforms differing significantly in abundance in control (Ctrl; blue) and HCM (red) tissue. Mono-phosphorylation is denoted by red p. Extracted ion chromatograms (EICs) generated using peaks from the top 5 charge states for each protein, with the area under the curve (AUC) highlighted (right, y axis is normalized to the EIC with the highest signal intensity), showing relative protein expression in each cohort. **C** Total phosphorylation (Ptotal) for control (blue) and HCM (red) cohort. **D** Quantitation of representative sarcomere protein expression by AUC for control (blue) and HCM (red) cohort. *n* = 12 for both cohorts. All plots show whiskers from 0 to 25th percentile, box from 25 to 75th percentile (mean indicated with line), and whiskers from 75 to 100th percentile. Differences between groups were considered statistically significant at an adjusted (adj.) *P* < 0.05, based on the Benjamini–Hochberg method for controlling the false discovery rate. *Adj. *P* < 0.05, **adj. *P* < 0.01. Certain sarcomeric proteins exist in multiple isoforms; therefore, protein names are displayed throughout the figure. +Phospho indicates proteoforms resulting from protein phosphorylation; α-CAA, cardiac alpha-actin; cTnI, cardiac troponin I; cTnT, cardiac troponin T; ENH2, enigma homolog isoform 2; FHL2, four and a half LIM domain protein 2; MLC-1v, myosin light chain 1; MLC-2v, myosin light chain 2v; MLP, muscle LIM protein; MYH7, beta-myosin heavy chain; N.S., not significant; TnC, troponin C; and Tpm1.1, α-tropomyosin. Figure and legend adapted from reference 50

**Fig. 6:**
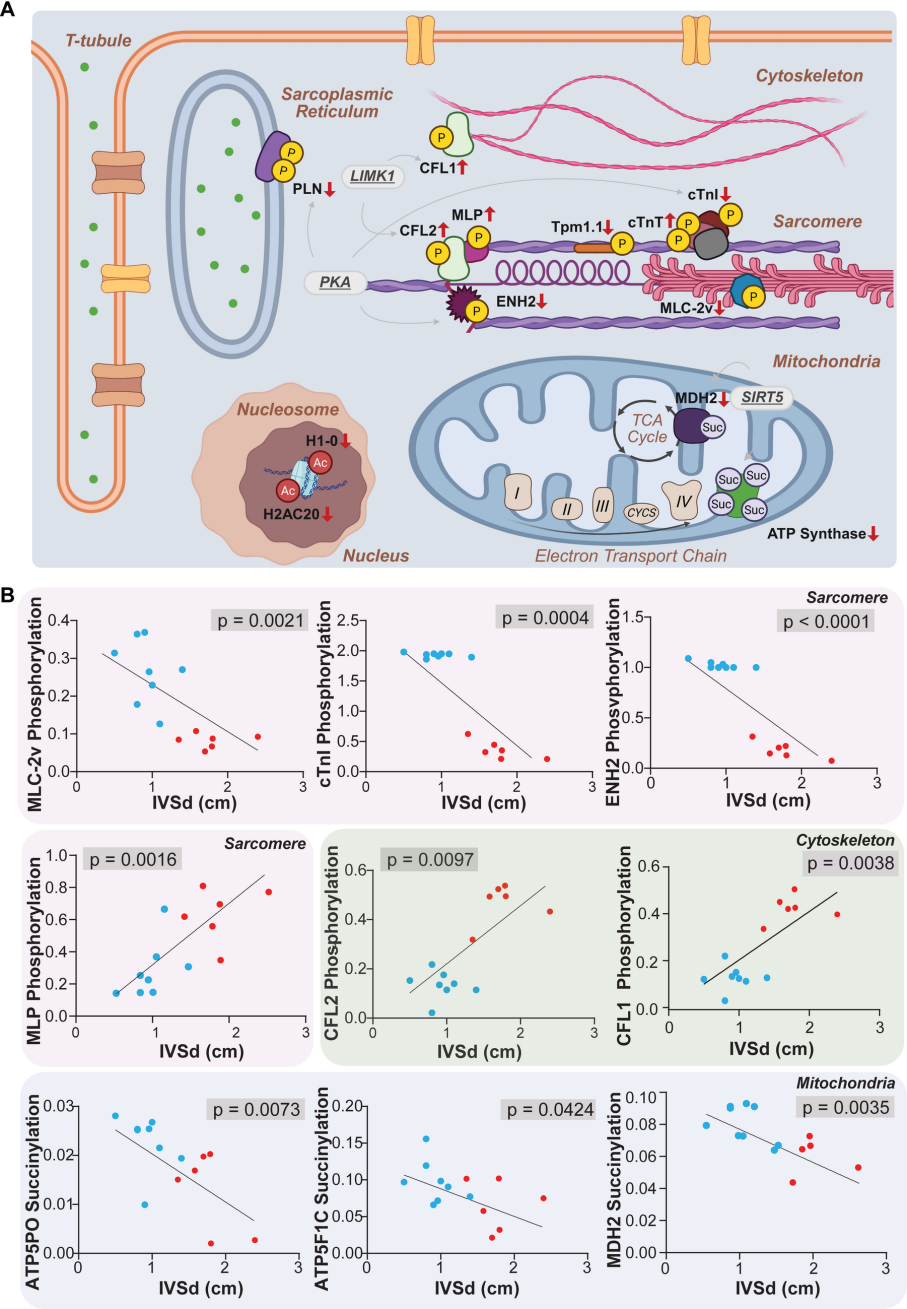
Global dysregulation of proteoforms across multiple cellular compartments underlines obstructive hypertrophic cardiomyopathy (HCM). **A** Key proteoform alterations were highlighted including altered phosphorylated proteoforms across the cardiac sarcomere, sarcoplasmic reticulum, and cytoskeleton, as well as reduced succinylated proteoforms in the mitochondria, and decreased acetylated proteoforms in the nucleosome. **B** Spearman correlations of clinical features. Abbreviations are as follows: IVSd, interventricular septal diameter. *P*-value determined by rank-based correlation for HCM (red) and control (blue) samples combined. Red arrows indicate increased or decreased PTM in HCM cohort. Figure and legend adapted from reference 50

Given the growing recognition of contributions from proteins outside the sarcomere to disease heterogeneity in HCM, we recently sought to more comprehensively characterize the proteoform alterations occurring in the HCM heart. Employing a sequential extraction strategy, with an MS-compatible photo-cleavable surfactant developed by our lab (Brown et al. 2019), we were able to expand coverage to include approximately 2000 proteoforms (Gao et al. 2025). The detected proteoforms originated from proteins that localize to disparate cellular compartments, such as the sarcomeres, sarcoplasmic reticulum, cytoskeleton, mitochondria, and nucleus (Gao et al. 2025). Quantitative TDP analysis confirmed decreased phosphorylation of cTnI, αTpm, and MLC-2v, as well as increased cTnT phosphorylation in HCM (Fig. 5) (Gao et al. 2025). Moreover, the greater proteome coverage afforded by the novel approach also allowed the detection of proteoforms of muscle LIM protein (MLP), a critical component of the Z-disc stretch sensing complex (Knöll et al. 2002). Surprisingly, we found that MLP phosphorylation was significantly increased in the advanced obstructive HCM patient myocardium compared to nonfailing controls (Fig. 5) (Gao et al. 2025). Outside the sarcomeres, proteoform differences were detected in the sarcoplasmic reticulum protein phospholamban, as well as two actin-binding proteins, cofilin-1 and cofilin-2 (Gao et al. 2025). The detected proteoform differences translated to significantly decreased and increased phosphorylation for phospholamban and cofilin-1/−2, respectively, in the HCM heart (Fig. 6) (Gao et al. 2025). Phospholamban is another well-known target of the PKA signaling pathway and the concerted reduction across cTnI, ENH2, and phospholamban further implicates dysregulation of this signaling pathway in advanced HCM (Fig. 6) (Gao et al. 2025).

A variety of mitochondrial proteoforms were detected with several resulting from succinylation (Gao et al. 2025). Intriguingly, the succinylation of mitochondrial proteins, including mitochondrial malate dehydrogenase (MDH2) and several components of the mitochondrial ATPase (ATP5F1C, *ATP5IF1*, ATP5PD, and ATP5PO), was decreased in the hearts of HCM patients (Fig. 6) (Gao et al. 2025). Succinylation, a PTM wherein a succinyl group is added to the ε-amino group of lysine residues, has been shown to play an important role in regulating mitochondrial enzyme activity (Rardin et al. 2013). This modification is primarily regulated by an enzyme called SIRT5 in the mitochondria and studies have shown that loss of SIRT5 in mice leads to the development of an HCM-like phenotype (Sadhukhan et al. 2016). Conversely, mice overexpressing SIRT5 have been shown to be resistant to maladaptive remodeling associated with transverse aortic constriction (Guo et al. 2022)—a well-established model for inducing hypertrophy and HF in rodents. Prior evidence indicates that protein succinylation is changed in ischemic cardiomyopathy in humans (Ali et al. 2020), and our findings, for the first time, expand changes in mitochondrial protein succinylation to human HCM (Gao et al. 2025).

We also detected a myriad histone proteoforms arising from single and combinatorial PTMs, including methylation, phosphorylation, and acetylation, in control and HCM patient tissues (Gao et al. 2025). Detected histone isoforms included representatives of all four core histone families (H2A, H2B, H3, and H4), as well as two isoforms of the H1 linker histone (Gao et al. 2025). These results highlight the power of TDP for the analysis of heavily modified proteins like histones by allowing for the unbiased and simultaneous detection of multiple PTMs. Even though the low abundance of histones in the TFA extract did not allow for extensive characterization or localization of histone PTMs, a global decrease in the acetylation of the H2AC20 core and H1-0 linker histones was detected in HCM patient tissues (Fig. 6) (Gao et al. 2025). In general, increased histone acetylation is associated with gene activation because of alterations in chromatin structure and/or via the recruitment of regulatory acetyllysine reader proteins (Shvedunova and Akhtar 2022). Histone acetylation is known to play an important role in epigenetic remodeling in cardiac hypertrophy (Backs and Olson 2006). Reinforcing this association, genetic manipulation of many of the genes encoding enzymes that regulate histone acetylation leads to either physiological or pathological hypertrophy of the myocardium (Qin et al. 2021; Wu et al. 2024). While the observed decrease in global H1-0 and H2AC20 acetylation is intriguing, it is important to note that further information, including PTM site information, as well as information regarding changes in histone modifications in the local environment of relevant genes, will be imperative for understanding how changes in epigenetic regulation contribute to HCM. Collectively, our findings provide the first broad view of proteoform alterations in HCM and demonstrate that proteoform alterations in multiple cellular compartments beyond the sarcomere contribute to the pathobiology of advanced-stage HCM (Gao et al. 2025).

### BUP—insights into metabolic dysfunction and hypertrophic pathway activation

Energetic defects are a hallmark of HCM with studies reporting a decreased ratio of phosphocreatine to ATP in hearts from patients with HCM versus controls (Crilley et al. 2003; Valkovič et al. 2019). It has been assumed that this results from inefficient sarcomeric ATP utilization that compromises the ability to maintain the energy levels necessary for other critical cellular functions in HCM (Ashrafian et al. 2003). Recent BUP studies have provided new insights into metabolic dysfunction in HCM. Previs et al. carried out paired proteomic and metabolomic analysis of surgical myectomy samples from three HCM patient genotypes (genotype-negative, *MYBPC3*, and *MYH7*) and septal tissue from nonfailing controls (Previs et al. 2022). Their comparison identified 88 proteins that differed in at least 1 HCM group and 57 proteins in all three HCM groups compared with control hearts (Previs et al. 2022). Among the differentially expressed proteins, there was a marked downregulation of mitochondrial and metabolic proteins consistent with profound energetic impairment (Previs et al. 2022). Proteins involved in mitochondrial fatty acid β-oxidation, including very long chain acyl-CoA dehydrogenase *ACADV*, hydroxyacyl-CoA dehydrogenase, and acetyl-CoA acyltransferase 2, were significantly down-regulated in HCM relative to nonfailing controls (Previs et al. 2022). Moreover, a 43% reduction in creatine-kinase M-type, which reversibly catalyzes the transfer of phosphate between ATP and creatine phosphate, was also detected in the HCM hearts (Previs et al. 2022). The proteomic evidence of mitochondrial dysfunction aligned with reduced components of oxidative metabolism and was supported by metabolomic depletion of long-chain acyl-carnitines, acetyl-CoA, ATP, and phosphocreatine as determined by paired metabolomic analysis (Previs et al. 2022). Notably, similar to our finding that proteoform alterations were consistent across patient HCM genotypes, the findings of Previs et al. were also independent of HCM genotype and sex (Previs et al. 2022). Their findings shift the view of metabolic insufficiency in HCM from energy depletion resulting from a failure to adapt energy production to meet the increased demand of hypercontractile sarcomeres to one wherein a loss of mitochondrial oxidative capacity and energy reserve at the protein level drive a metabolic shift away from efficient fatty acid oxidation toward alternative, less energetically favorable substrates (Previs et al. 2022). This work suggests that ATP production itself is defective in the HCM heart and that the mitochondria are critical players in energetic insufficiency.

Garmany et al*. *carried out BUP analysis of a subset of individuals from a large HCM cohort with 43 genotype-positive and 33 genotype-negative individuals (Garmany et al. 2023). Consistent with the findings of other studies (Previs et al. 2022), including our own (Tucholski et al. 2020), their results revealed that genotype-positive HCM did not separate by principal component analysis from genotype-negative HCM indicating that the proteome of HCM is similar across patient genotypes (Garmany et al. 2023). Comparison of the HCM and nonfailing proteomes identified ~411 proteins (~9% of quantified proteome) differentially expressed in HCM, pointing to broad proteome remodeling with notable differences in proteins involved in metabolism and hypertrophic signaling (Garmany et al. 2023). Notably, BUP analysis identified upregulation of six canonical hypertrophic pathways (ERK5 signaling, ERK/MAPK signaling, HER-2 signaling, endothelin-1 signaling, PI3K/AKT signaling, and UVA-induced MAPK signaling), five of which showed downregulation at the transcript level in paired analysis (Garmany et al. 2023). Findings such as this reinforce the need for protein-level analysis in complex diseases such as HCM. Phosphoproteomic analysis identified 372 and 880 proteins that were hypo- and hyper-phosphorylated, respectively, in HCM versus nonfailing control (Garmany et al. 2023). Among the hyperphosphorylated proteins were several components of the RAS-MAPK pathway (Garmany et al. 2023). Given that the hypertrophic pathways activated in HCM hearts converged on the RAS-MAPK signaling cascade, these findings suggest this as a final common pathway in late-stage HCM (Garmany et al. 2023).

## Concluding remarks and perspective

The longstanding paradigm of HCM as the archetypal monogenic disease caused by pathogenic variants in the genes encoding constituents of the sarcomere is inadequate to explain the clinical and biological heterogeneity observed in affected patients. As highlighted in this mini-review, recent MS-based proteomics studies provide compelling evidence that HCM is characterized by extensive molecular remodeling that spans multiple cellular compartments, including the sarcomeres, sarcoplasmic reticulum, cytoskeleton, mitochondria, and nucleus. Importantly, these proteomic alterations are remarkably consistent across genotype-positive and genotype-negative patients, suggesting that advanced HCM converges on shared downstream molecular phenotypes irrespective of the initiating genetic insult.

TDP studies from our lab have revealed coordinated dysregulation of proteoforms and PTMs in both sarcomeric and extra-sarcomeric proteins, implicating altered kinase signaling, mitochondrial protein regulation through succinylation, and epigenetic remodeling in disease pathogenesis. Complementary BUP studies have further demonstrated broad suppression of mitochondrial oxidative metabolism as a primary source of metabolic insufficiency in HCM and activation of hypertrophic signaling pathways. Together, these findings underscore the importance of protein-level regulation in shaping the complex clinical phenotype of HCM.

Looking forward, top-down proteomics is uniquely positioned to advance our understanding of HCM pathobiology and bridge the gap between clinical phenotype and patient genotype. Continued application of high-resolution proteomic technologies, particularly in combination with multi-omics and spatially resolved approaches, will be essential for defining disease modifiers, identifying therapeutic targets beyond the sarcomere, and ultimately improving precision medicine strategies for patients with HCM.

## Data Availability

No datasets were generated or analyzed during the current study.
